# Poor CD4/CD8 ratio recovery in HBcAb-positive HIV patients with worse immune status is associated with significantly higher CD8 cell numbers

**DOI:** 10.1038/s41598-021-83616-z

**Published:** 2021-02-17

**Authors:** Vincenzo Malagnino, Carlotta Cerva, Elisabetta Teti, Laura Campogiani, Mirko Compagno, Luca Foroghi Biland, Laura Saderi, Daniele Armenia, Romina Salpini, Valentina Svicher, Giovanni Sotgiu, Marco Iannetta, Massimo Andreoni, Loredana Sarmati

**Affiliations:** 1 Clinic of Infectious Diseases, Policlinico Tor Vergata, V. Montpelier 1, 00133 Rome, Italy; 2 Department of Systems Medicine, Tor Vergata University of Rome, Rome, Italy; 3 Department of Experimental Medicine and Surgery, Tor Vergata University of Rome, Rome, Italy; 4 Department of Medical, Surgical and Experimental Sciences, University of Sassari, Sassari, Italy; 5 Saint Camillus International, UniCamillus, University of Health Sciences, Rome, Italy

**Keywords:** Viral hepatitis, HIV infections, CD8-positive T cells

## Abstract

Low CD4+ cell count in patients with human immunodeficiency virus (HIV) and hepatitis B virus (HBV) coinfection during combination antiretroviral therapy (cART) has been described; however, notably few studies have investigated coinfected patients positive for antibodies to the HBV c antigen (HBcAb). An observational retrospective study enrolling 190 patients was conducted by grouping patients with respect to HBV status and recording CD4+ T cell counts and percentages (CD4%), CD8+ T cell counts and percentages (CD8%), and the CD4+ to CD8+ T cell ratio (CD4/CD8) at the time of HIV diagnosis, at the start of treatment and at months 1, 2, 3, 4, 5, 6, 12, and 24 after beginning cART. One hundred and twenty patients (63.2%) were negative for previous HBV infection, while 70 (36.8%) were HBcAb-positive. A significant increase in the CD4/CD8 ratio was recorded in HIV monoinfected subjects compared to HBV coinfected patients from months 4 to 12 from the beginning of cART (*p* value = 0.02 at month 4, *p* value = 0.005 at month 5, *p* value = 0.006 at month 6, and *p* value = 0.008 at month 12). A significant increase in the absolute count of CD8+ T lymphocytes was described from months 2 to 24 from the start of cART in the subgroup of HBV coinfected patients with an AIDS event at the onset of HIV infection. The presence of HBcAb was observed to be associated with reduced CD4/CD8 ratio growth and a significantly higher proportion of subjects with CD4/CD8 < 0.45 in the HIV/HBV coinfected group. A significant increase in the CD8 T cell count was shown up to 24 months after the initiation of effective cART in the subgroup of patients with the worst immune status.

## Introduction

Hepatitis B virus (HBV) infection is associated with increased mortality for liver-related diseases in human immunodeficiency virus (HIV)-positive patients^1,2^, and chronic HBV infection (CHB) is related to a more rapid progression of HIV disease^3–5^.

The presence of isolated hepatitis B core antibody (HBcAb) is a sign of poor immune control against HBV and is considered a surrogate marker of occult HBV infection (OBI). OBI is characterized by the persistence of HBV DNA in the liver, regardless of whether HBV DNA is detectable in serum or not. OBI has been documented to occur more frequently in individuals from areas of high HBV endemicity and/or in immunocompromised patients. A prevalence of OBI ranging from 1 to 88% has been reported in HIV patients^6^, and cases of HBV reactivation have been reported in HIV-positive patients with OBI^7^.

OBI in HBV monoinfected patients is recognized as a risk factor for the progression to liver fibrosis, end-stage liver disease (ESLD) and hepatocellular carcinoma (HCC)^8–10^, though little is known about the clinical relevance of OBI in HIV-infected patients. Wandeler et al. and Cohen and colleagues^11,12^ showed that CD4 cell recovery was significantly impaired in hepatitis B s antigen (HBsAg)-positive and isolated HBcAb-positive HIV-positive subjects. In addition, Anderson et al. described an association between an increase in CD4 cell counts and HBV load suppression^13^. However, other studies did not report any association between HBV serostatus and immunological response to cART^14–18^.

We recently showed that HBcAb-positive status resulted in a delay in achieving HIV < 50 copies/mL and in the appearance of viral rebound during the course of cART, thus correlating poor control of HIV replication to the presence of HBcAb^19^. The aim of this study was to evaluate the CD4 and CD8 cell counts and the CD4/CD8 ratio at different times after the initiation of cART in the same group of HIV-HBcAb-positive and HIV-HBcAb-negative patients.

## Materials and methods

### Study design

The study population was described in a previous publication^19^. Briefly, an observational retrospective study recruiting 671 HIV-positive patients was performed from January 2007 to July 2018 at the Infectious Diseases Unit of the Policlinico Tor Vergata in Rome, Italy. The following data were collected at baseline: haematochemical variables, HBV and hepatitis C (HCV) serology, HIV-RNA viral load, date of HIV infection diagnosis and date of cART initiation. Data on flares of transaminases (i.e., aspartate aminotransferase [AST] or alanine aminotransferase [ALT] > 50 UI/L), HIV viral blips (VB, single detection of HIV-RNA > 50 cp/mL), virological failure (VF, two consecutive HIV-RNA ≥ 50 cp/mL or one > 1000 cp/mL with consequent change of cART resulting in virological suppression) and time to achieve viral undetectability (virological success [VS], two consecutive HIV-RNA < 50 cp/mL) were collected during follow-up.

Data on CD4+ T cell counts and percentages (CD4%), CD8+ T cell counts and percentages (CD8%), and the CD4+ to CD8+ T cell ratio (CD4/CD8) were collected at the following defined time points: the time of HIV diagnosis, start of cART and at months 1, 2, 3, 4, 5, 6, 12, and 24 after the beginning of cART. Laboratory measurements were carried out in accordance with good clinical practices indicated by national and international guidelines on the treatment of patients with HIV infection^20,21^.

The study protocol and related informed consent were submitted to and approved by Comitato Etico Indipendente at La Fondazione PTV Policlinico Tor Vergata [Protocol Number 216/16, version 1.0], and the study protocol was structured with respect to privacy. Personal information was treated in a confidential manner, and clinical data were anonymized in accordance with the Helsinki Declaration (version October 2013). All included patients signed informed consent for inclusion in the observational study.

### Inclusion and exclusion criteria

**Figure 1:**
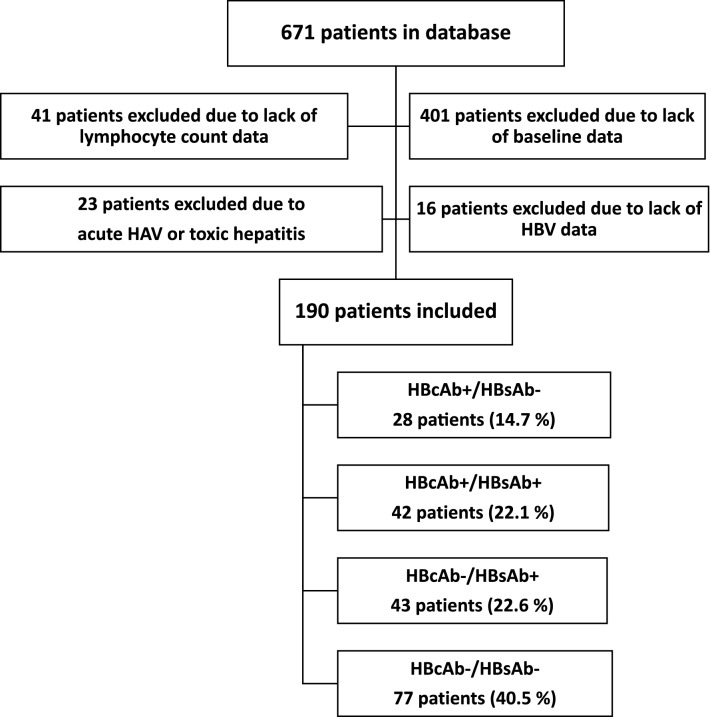
Study population inclusion and exclusion criteria algorithm.

Four hundred and one of 671 patients were excluded because of missing virological or immunological data at the time of diagnosis (i.e., non-adherence to follow-up visits, transfer from other clinical centres, and patients who voluntarily stopped their cART). Sixteen were excluded because of missing HBV serology data, 23 because of transaminase flares associated with hepatic drug toxicity (n = 2) and acute hepatitis A virus infection (n = 21), and 41 because of missing lymphocyte counts during follow-up. One hundred and ninety patients were ultimately included for analysis from the diagnosis of HIV infection to 24 months post-cART (Fig. 1).

All enrolled patients had achieved virological suppression following the initiation of first-line antiretroviral therapy.

### Endpoints

The primary endpoint was to evaluate the impact of HBcAb positivity on CD4+ and CD8+ cell counts and CD4/CD8 T lymphocyte ratio improvement at different time points (1, 2, 3, 4, 5, 6, 12 and 24 months) after the start of cART. Immunological success (IS) was defined as an increase of 150 CD4+ cells/mmc within 24 months after the start of cART^20,22^.

### Laboratory testing for the diagnosis of HBV and HIV infections

HBV serological markers were measured using immune-enzymatic assays (Roche/Cobas Diagnostics, Rotkreuz, Switzerland). Plasma HBV-DNA was detected using real-time polymerase chain reactions (20 IU/mL lower limit of quantification) (Roche/Cobas Ampliprep/Cobas Taqman, Rotkreuz, Switzerland). Plasma HIV-RNA levels were measured using a commercial test with 20 cp/mL HIV-RNA as the lower limit of quantification (COBAS AmpliPrep/COBAS TaqMan HIV-1 Test, v2.0), as described in a previous study^19^.

### Laboratory testing for lymphocyte subpopulations

T lymphocyte subset absolute counts were obtained after labelling whole blood samples with BD Multitest 6-Colour TBNK Reagent using BD Trucount™ tubes (Becton Dickinson, San Jose, CA, USA). A lyse-no-wash protocol was used. Briefly, whole blood was stained with the Multitest 6-colour TBNK reagent (containing a cocktail of the following antibodies: CD3 FITC, CD16 and CD56 PE, CD45 PerC*p* Cy5.5, CD4 PE-Cy7, CD19 APC, and CD8 APC-Cy7). After the first incubation (15 min at room temperature in the dark), BD FACS lysing solution (Becton Dickinson, San Jose, CA, USA) was added to the tubes. After a second incubation (15 min at room temperature in the dark), samples were run on a BD FACSCanto II and analysed with the BD FACSCanto clinical software (Becton Dickinson, San Jose, CA, USA).

### Statistical methods

An Excel database created for the previous study^19^ was used to collect all study variables. Qualitative data are summarized with absolute and relative frequencies (percentages). Means and standard deviations (SD) or medians and interquartile ranges (IQR) were used to describe quantitative data with a normal or nonnormal distribution, respectively. Chi-squared or Fisher exact tests were used to detect any significant differences in the comparison of qualitative variables. Student’s t- and Mann–Whitney tests were performed to compare quantitative variables between the HBcAb-negative and HBcAb-positive groups in the case of parametric and nonparametric distributions, respectively. ANOVA and Kruskal–Wallis tests were used to detect any significant differences in the comparison of parametric and nonparametric quantitative variables, respectively, between the HBcAb−, HBcAb+  /HBcAb− and HBcAb+  /HBsAb + groups. Comparison of CD4+ counts during follow-up was performed with Friedman's test. A two-tailed *p* value less than 0.05 was considered statistically significant. Stata 16 (College Station, TX: StataCorp LLC) and IBM SPSS Statistics version 23 were used for statistical computations.

## Results

### Study population

The study sample characteristics are shown in Table 1. A total of 190 patients were enrolled; their median (IQR) age was 42 (31–52) years and 81.6% were Caucasian. The majority (89.5%) acquired HIV infection through sexual intercourse. The median follow-up period was 4.3 years (1574 days) and the median duration of cART was 3.8 years (1405 days, IQR 976–2219).

**Table 1:** Descriptive analysis.

	n = 190
**Median (IQR) age, years**	42 (31–52)
**Origin, n (%)**	
Caucasian	155 (81.6)
African	10 (5.3)
East Europe	21 (11)
Asia	1 (0.5)
Other	3 (1.6)
**Risk factors, n (%)**	
Sexual	170 (89.5)
Injecting drug users	20 (10.5)
**Median (IQR) duration of cART, days**	1405 (976–2219)
**Median (IQR) follow-up, days**	1574 (1024–2436)
**cART, n (%)**	
2NRTI + PI	84 (44.2)
2NRTI + nnrti	53 (27.9)
2NRTI + INI	45 (23.7)
Other	8 (4.2)
**HBV serology, n (%)**	
Negative	77 (40)
HBcAb positive	70 (37)
HBcAb	28 (40)
HBcAb+ /HBsAb +	42 (60)
HBV vaccination	43 (23)
**HCV antibody positivity, n (%)**	9 (4.8)
**Median (IQR) CD4 cell count at baseline, cells/mmc**	237 (102–419)
**Median (IQR) nadir) CD4 cell count**	209 (85–357)
**CD4/CD8 T lymphocyte ratio < 0.45 at baseline, n (%)**	117 (61.6)
**Median (IQR) HIV-RNA at baseline, cp/mL**	84,932 (34,314–292,310)
**Mean (SD) log HIV-RNA at baseline, cp/mL,**	4.9 (0.7)
**Undetectability after 6 months of cART**	146 (76.8)
**Median (IQR) undetectability, months**	5 (3–6)
**AIDS-related event, n (%)**	48 (25.3)
**Non-AIDS related event, n (%)**	75 (39.5)
**VF, n (%)**	26 (13.7)
**VB, n (%)**	82 (43.2)

VB = viral blip, single detection of HIV-RNA > 50 cp/mL; VF = viral failure, two consecutive HIV-RNA ≥ 50 cp/mL or one > 1000 cp/mL with consequent change of cART.

cART = combined antiretroviral therapy; NRTI = nucleoside/nucleotide reverse transcriptase inhibitor; NNRTI = nonnucleoside reverse-transcriptase inhibitors; PI = protease inhibitors; undetectability = HIV-RNA < 50 copies/mL.

AIDS-related events = the occurrence of any opportunistic infections or malignancy according to the World Health Organization (ref. 20); non-AIDS-related events = comorbidities such as hypertension, diabetes and lipidic disorders, bone disorders (osteopenia, osteoporosis), renal impairment and non-AIDS-related neoplastic pathologies.

The median (IQR) CD4+ lymphocyte counts at baseline and CD4+ cell nadir were 237 (102–419) cells/mmc and 209 (85–357) cells/mmc, respectively. One hundred and seventeen (61.6%) patients had a baseline CD4/CD8 lymphocyte ratio < 0.45. The mean baseline HIV viremia was 4.9 log cp/mL. Six months after the beginning of cART, 146 (76.8%) patients achieved an HIV viremia < 50 cp/mL. During follow-up, 26 (13.7%) patients experienced VF and 82 (43.2%) experienced VB.

Seventy (36.8%) patients were HBcAb-positive, of whom 42 (60%) were also HBsAb-positive. One hundred and twenty patients (63.2%) were negative for HBV infection, 77 (40.5%) were negative for all HBV infection markers, and 43 (22.6%) were vaccinated and positive for only HBsAb.

### CD4+ and CD8+ T lymphocyte numbers and CD4/CD8 ratio in the 24 months after cART initiation in the two subgroups of HIV-positive/HBcAb-negative and HIV-positive/HBcAb-positive patients

**Figure 2:**
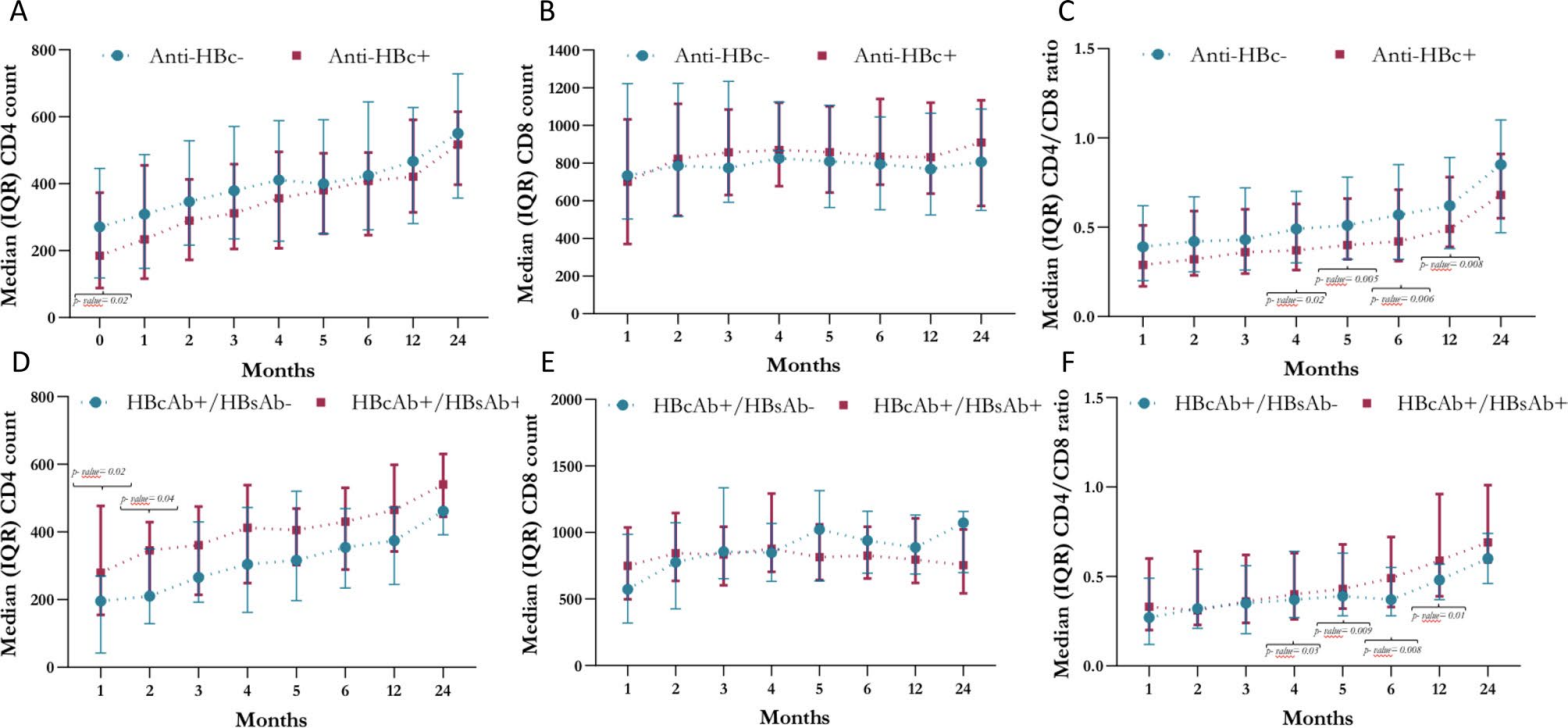
Comparison between CD4+ and CD8+ T cell numbers and CD4/CD8 ratio improvement in the 24 months following the start of cART in a population of HBcAb+  and HBcAb- HIV-positive subjects (**A**, **B** and **C**, respectively) and of HBcAb+  /HBsAb- and HBcAb+  /HBsAb + HIV-positive subjects (**D**, **E** and **F**, respectively).

Differences between HBcAb-positive and HBcAb-negative HIV patients are shown in Fig. 2A-C and in Table S1. Reduced CD4+ T lymphocyte absolute counts were found in the HBcAb-positive group before starting cART (baseline: 185 cells/mmc [88–373] vs. 271 cells/mmc [118.5–445.5], *p* = 0.02; in the HBcAb-positive group vs. HBcAb-negative group, respectively) (Fig. 2A). With respect to CD4/CD8 ratio values (Fig. 2C), no difference was noted between the two patient groups up to 3 months after cART initiation. From 4 to 12 months, a significantly higher CD4/CD8 ratio was observed in HIV monoinfected subjects than in HBcAb+  HIV+ individuals (month 4: 0.49 [0.3–0.7] vs. 0.37 [0.26–0.63], *p* value = 0.02; month 5: 0.51 [0.32–0.78] vs. 0.4 [0.32–0.66], *p* value = 0.005; month 6: 0.57 [0.32–0.85] vs. 0.42 [0.31–0.71], *p* value = 0.006; and month 12: 0.62 [0.38–0.89] vs. 0.49 [0.39–0.78], *p* value = 0.008).

Similarly, the percentage of patients with a CD4/CD8 ratio < 0.45 was significantly higher in HBV coinfected patients at 2–12 months from cART initiation than in HIV monoinfected individuals (month 2: 68.6% vs. 54.2% *p* = 0.05; month 3: 67.1% vs. 50.8%, *p* = 0.03; month 4: 65.7% vs. 39.2% *p* value < 0.0001; month 5: 64.3% vs. 38.3%, *p* value = 0.001; month 6: 57.1% vs. 35.8%, *p* value = 0.004; and month 12: 47.1% vs. 32.5%, *p* value = 0.05, for the HBcAb-positive group vs. HIV monoinfected group, respectively) (Table S1).

No other differences were documented between the 2 patient groups, and both groups achieved IS 24 months post-cART initiation.

### CD4+ and CD8+ T lymphocyte numbers and CD4/CD8 ratio improvement in the 24 months after cART initiation in the two subgroups of HIV/HBcAb-positive patients with and without HBsAb positivity

With the aim of assessing whether the presence of HBsAb in HIV/HBcAb-positive individuals identified patients with a better immunological status, a separate evaluation of CD4+ and CD8+ T lymphocyte absolute counts and the CD4/CD8 ratio rise was performed in the subsets of HBcAb-positive/HBsAb-positive and HBcAb-positive/HBsAb-negative coinfected patients, and the results are shown in Fig. 2D-F and Table S2. A significantly higher CD4 lymphocyte count was observed at months 1 and 2 post-cART initiation in the HBcAb-positive/HBsAb-positive group than in the HBcAb-positive/HBsAb-negative group (1 month: 279.5 cells/mmc [155–477] vs. 195.5 cells/mmc [42.5–269.5], *p* value = 0.02; 2 months: 345.5 cells/mmc [207–429] vs. 210 cells/mmc [129–349.5], *p* value = 0.04 in the HBsAb-positive group vs. HBsAb-negative group, respectively) (Fig. 2D). Similarly, when compared to HBcAb-positive/HBsAb-negative patients, HBcAb-positive/HBsAb-positive patients exhibited significantly higher CD4/CD8 T cell ratio values from 4 to 12 months, while there was a lower percentage of subjects with a CD4/CD8 ratio < 0.45 from 3 to 12 months after cART initiation (CD4/CD8 ratio, month 4: 0.4 [0.26–0.63] vs. 0.37 [0.27–0.64], *p* value = 0.03; month 5: 0.43 [0.32–0.68] vs. 0.39 [0.28–0.63], *p* value = 0.009; month 6: 0.49 [0.33–0.72] vs. 0.37 [0.28–0.55], *p* = 0.008; and month 12: 0.59 [0.39–0.96] vs. 0.48 [0.37–0.57], *p* value = 0.01) (Fig. 2F) (CD4/CD8 ratio < 0.45, month 3: 61.9% vs. 75% *p* value 0.05; month 4: 61.9% vs. 71.4% *p* value < 0.001; month 5: 59.5% vs. 71.4% *p* value = 0.002; month 6: 50% vs. 67.9%, *p* value = 0.006; and month 12: 38.1% vs. 60.7%, *p* value = 0.02 for the HBsAb-positive vs. HBsAb negative group, respectively) (Table S2).

### CD4+ and CD8+ T lymphocyte numbers and CD4/CD8 ratios in the 24 months after cART initiation in HBcAb-positive and HBcAb-negative patients with an AIDS event at HIV infection diagnosis (AIDS-presenting patients)

To evaluate whether the condition of severe immunodeficiency influenced the CD4 cell number and CD4/CD8 ratio improvement in the HBcAb-positive and HBcAb-negative groups, the subgroup of patients in which the diagnosis of HIV seropositivity was made at the same time as an AIDS-defining condition was analysed separately (Table 2). An AIDS clinical event was defined as the occurrence of any opportunistic infections or malignancy according to the World Health Organization (20). No significant differences in terms of CD4 T lymphocyte absolute counts, CD4/CD8 ratio, percentage of patients with a CD4/CD8 ratio < 0.45 or rate of subjects achieving IS were found between 20 HBcAb-negative and 28 HBcAb-positive patients 24 months after cART initiation. A significant increase of the absolute count of CD8+ lymphocytes was observed in the group of HBV coinfected patients from 2 to 24 months after cART initiation (month 2: 661.5 [393–1094] vs. 426 [258–671] *p* value = 0.03; month 3: 726.5 [538–1291] vs. 430.5 [328.5–699.0], *p* value = 0.01; month 4: 857 [562–1434] vs. 523.5 [356.5–757], *p* value = 0.006; month 5: 825 [636–1327] vs. 493 [359.5–741.5], *p* value = 0.008; month 6: 822 [598–1180] vs. 466.5 [368.5–765.0], *p* value = 0.007; month 12: 831.5 [621–1079] vs. 559.5 [382.0–899.5], *p* value = 0.01; and month 24: 1038 [530–1239] vs. 700 [450–990], *p* value = 0.05, for the HBcAb-positive group vs. HBcAb-negative group, respectively).

**Table 2:** Comparison between CD4+ and CD8+ T lymphocyte numbers and CD4/CD8 ratio improvement in the 24 months following the start of cART in the following two AIDS-presenting patient subgroups: HBcAb-positive and HBcAb-negative.

	AIDS patients/HBcAb- (n = 20)	AIDS patients/HBcAb+ (n = 28)	*p* value
Median (IQR) CD4 count, 1st month	96 (14.5–130.5)	143 (53–265)	0.12
Median (IQR) CD4 count, 2nd month	89.5 (62.0–275.5)	202.5 (104.5–305.0)	0.15
Median (IQR) CD4 count, 3rd month	137.5 (91.5–193.0)	212 (137.5–361.0)	0.08
Median (IQR) CD4 count, 4th month	134.5 (105.5–257.0)	211 (158–360)	0.09
Median (IQR) CD4 count, 5th month	172 (135.0–259.5)	254 (139–410)	0.09
Median (IQR) CD4 count, 6th month	165 (118.5–262.0)	323 (118–408)	0.18
Median (IQR) CD4 count, 12th month	237 (146.0–296.5)	321.5 (160–465)	0.21
Median (IQR) CD4 count, 24th month	343 (227–521)	476 (272–551)	0.17
Median (IQR) CD8 count, 1st month	350 (233.5–533.0)	556 (273–811)	0.13
Median (IQR) CD8 count, 2nd month	426 (258–671)	661.5 (393–1094)	**0.03**
Median (IQR) CD8 count, 3rd month	430.5 (328.5–699.0)	726.5 (538–1291)	**0.01**
Median (IQR) CD8 count, 4th month	523.5 (356.5–757.0)	857 (562–1434)	**0.006**
Median (IQR) CD8 count, 5th month	493 (359.5–741.5)	825 (636–1327)	**0.008**
Median (IQR) CD8 count, 6th month	466.5 (368.5–765.0)	822 (598–1180)	**0.007**
Median (IQR) CD8 count, 12th month	559.5 (382.0–899.5)	831.5 (621–1079)	**0.01**
Median (IQR) CD8 count, 24th month	700 (450–990)	1038 (530–1239)	**0.05**
Median (IQR) CD4/CD8 ratio, 1st month	0.22 (0.08–0.40)	0.22 (0.07–0.42)	0.86
Median (IQR) CD4/CD8 ratio, 2nd month	0.28 (0.14–0.47)	0.28 (0.13–0.38)	0.79
Median (IQR) CD4/CD8 ratio, 3rd month	0.27 (0.14–0.55)	0.28 (0.10–0.42)	0.77
Median (IQR) CD4/CD8 ratio, 4th month	0.33 (0.17–0.50)	0.29 (0.11–0.39)	0.25
Median (IQR) CD4/CD8 ratio, 5th month	0.44 (0.16–0.50)	0.35 (0.14–0.40)	0.20
Median (IQR) CD4/CD8 ratio, 6th month	0.36 (0.18–0.60)	0.34 (0.12–0.47)	0.34
Median (IQR) CD4/CD8 ratio, 12th month	0.43 (0.24–0.60)	0.36 (0.21–0.52)	0.40
Median (IQR) CD4/CD8 ratio, 24th month	0.51 (0.30–0.68)	0.44 (0.24–0.79)	0.58
CD4/CD8 ratio < 0.45, 1st month, n (%)	17 (85.0)	23 (82.1)	0.79
CD4/CD8 ratio < 0.45, 2nd month, n (%)	15 (75.0)	22 (78.6)	0.77
CD4/CD8 ratio < 0.45, 3rd month, n (%)	14 (70.0)	24 (85.7)	0.19
CD4/CD8 ratio < 0.45, 4th month, n (%)	12 (60.0)	24 (85.7)	0.04
CD4/CD8 ratio < 0.45, 5th month, n (%)	12 (60.0)	23 (82.1)	0.09
CD4/CD8 ratio < 0.45, 6th month, n (%)	11 (55.0)	20.0 (71.4)	0.24
CD4/CD8 ratio < 0.45, 12th month, n (%)	10 (50.0)	18 (64.3)	0.32
CD4/CD8 ratio < 0.45, 24th month, n (%)	8 (40.0)	17 (60.7)	0.16
CD4/CD8 ratio recovery ≥ 0.45 at 24th month, n (%)	10 (58.8)	10 (43.5)	0.34
Immunological success at 24th month, n (%)	14 (70.0)	24 (85.7)	0.28

## Discussion

The present study demonstrated that HBcAb negatively affects CD4/CD8 ratio improvement during the first twelve months after cART initiation in a population of HIV/HBV coinfected patients, with a significantly higher number of HIV-HBcAb-positive subjects with a CD4/CD8 ratio < 0.45 up to the twelfth month from the start of therapy. Furthermore, HBcAb positivity was associated with a significant increase in CD8 T lymphocyte counts up to 24 months after the start of effective cART in the subgroup of patients with the worst immune status.

Impaired CD4 recovery during cART in HIV patients with CHB has been described^23–26^. This phenomenon has been attributed to immune activation and T cell apoptosis dependent on active HBV replication, as demonstrated by the lower CD4 T lymphocyte counts in cART-treated coinfected patients^13^.

Notably, few studies have investigated HIV patients with resolved HBV infection (HBcAb positivity). A study of 508 Chinese HIV subjects showed that the HBcAb-positive group (13.6%) had a significantly lower median CD4 cell count than HIV monoinfected patients at the start of cART^4^. Moreover, a low CD4 cell count was primarily attributed to Occult B Infection (OBI) (~ 10%) in a large cohort of isolated HBcAb-positive HIV-infected women^27^, and in the Swiss HIV Cohort Study^11^, higher CD4 cell recovery was reported in HBV-uninfected patients during the first 3 years of cART. The detection of anti-HBc in the blood represents a waning host immune response to HBV infection^28^ and is considered a surrogate marker of OBI in immunocompromised patients^29^. HBV reactivation was frequently reported in AIDS patients with CHB or resolved HBV infection (HBcAB positivity), so the American Guidelines for antiretroviral therapy^30^ recommend HBV vaccines in isolated HBcAb-positive patients with HIV infection to stimulate the HBsAb response to better control HBV replication. In a population of 191 cART-naive HIV- and HBcAb-positive subjects, Cohen demonstrated that OBI subjects (HBV DNA positivity 11.1%) had a lower number of CD4+ lymphocytes and HBV DNA was no longer detectable once cART was started^12^.

In our previous article, performed on the same patient population as the present study, we found poor control of HIV viremia 2 years after the start of cART in HBcAb-positive subjects and argued that occasional HBV replication may contribute to promoting HIV replication^19^. The slow growth of the CD4/CD8 ratio and the higher proportion of subjects with a low (< 0.45) CD4/CD8 ratio after starting cART demonstrated in the present study may be the result of impaired control of HIV replication due to the persistence of HBV viremia, with consequent prolonged CD8+ lymphocyte activation. The lack of HBV-DNA detection in the plasma of patients with OBI after the initiation of cART, in Cohen’s study, does not exclude the possibility of the presence of 'cryptic' HBV replication. The presence of HBV-DNA has been demonstrated with a highly sensitive droplet digital PCR assay in the liver of HBcAb-positive HBV-DNA plasma-negative donors^31^, and a series of new biomarkers has recently been used to detect HBV reactivation early in immunocompromised subjects^32^.

The presence of HBsAb appears to mitigate the negative effects of HBcAb on CD4/CD8 ratio improvement. HBsAb detection has been linked with more efficient antiviral T-cell responses and subsequently to viral neutralization^33^ and protection against HBV reactivation in immunocompromised patients who underwent chemotherapy^34^. Loss of HBsAb is associated with coinfections, including HIV, and is probably linked with a poor immune response; therefore, HBsAb might represent an indirect marker of a more efficient immune system.

Significant increases in CD8 T lymphocyte counts in HBV/HIV coinfection were observed in the group of patients with an AIDS event at onset. CD8 T cell counts are elevated during HIV infection and decreased slightly during cART^35^. CD8 proliferation was also demonstrated to occur in other viral infections (e.g., cytomegalovirus or hepatitis C virus)^36^. A study of the Collaboration of Observational HIV Epidemiological Research in Europe (COHERE) showed an increased risk of virological failure in HIV patients with high CD8 T cell counts after 5 years of cART^37^. Furthermore, Helleberg et al. reported that a CD8 cell count > 1500 cells/mmc was associated with increased mortality for non-AIDS events in HIV-positive patients after 10 years of cART^35^. The higher CD8 lymphocyte counts observed in HIV-HBcAb-positive patients up to 24 months from the start of cART may represent a marker of persistent CD8 T cell proliferation attributed to HBV replication. Furthermore, in HIV-infected adolescents, an elevated CD8 immune activation rate (as assessed by the increased percentages of CD8 cells expressing both CD38 and HLA-DR) has been associated with a decreased ability of the immune system to respond to HBV vaccination^38^. We can speculate that the immune dysregulation induced by HIV infection in AIDS-presenting patients can impair HBsAb production, allowing for possible HBV replication and further CD8 expansion.

This study has several limitations. The single-centre and retrospective properties of the study may cause selection bias. Unfortunately, data on HBV-DNA were not available. The lack of CD8+ T lymphocyte subset analysis may hamper the reliability of these conclusions.

The presence of HBcAb is associated with poor control of HIV infection^19^, an impaired CD4/CD8 ratio and a significantly higher CD8 T cell number during the first two years of cART. It is important to assess whether OBI negatively affects the immune system of HIV/HBV coinfected patients.

## Supplementary Information

Supplementary Information
