# Supplementary material for: Poor CD4/CD8 ratio recovery in HBcAb-positive HIV patients with worse immune status is associated with significantly higher CD8 cell numbers
Source: Sci Rep. 2021 Feb 17;11:3965. doi: 10.1038/s41598-021-83616-z (PMC7889897; doi:10.1038/s41598-021-83616-z)
Supplement: Supplementary file 1 — Supplementary Information [file 41598_2021_83616_MOESM1_ESM.docx]

S**upplementary Tables**

**Table S1. Comparison between CD4+ and CD8+ T lymphocyte numbers, CD4/CD8 ratio and CD4/CD8 ratio < 0.45 for 24 months following the start of cART in a population of HIV-infected HBcAb-negative and HBcAb-positive subjects.**

|  | **HIV+/HBcAb-**  **(n= 120)** | **HIV+/HBcAb+**  **(n= 70)** | **p-value** |
| --- | --- | --- | --- |
| *Median (IQR) CD4 count, baseline* | 271 (118.5-445.5) | 185 (88-373) | **0.02** |
| *Median (IQR) CD4 count, 1^st^ month* | 309 (147.5-487) | 233.5 (116-455) | 0.07 |
| *Median (IQR) CD4 count, 2^nd^ month* | 346 (216.5-528.5) | 289.5 (172-413) | 0.06 |
| *Median (IQR) CD4 count, 3^rd^ month* | 379 (235-571) | 331.5 (205-458) | 0.13 |
| *Median (IQR) CD4 count, 4^th^ month* | 411 (228.5-588.5) | 356 (207-495) | 0.26 |
| *Median (IQR) CD4 count, 5^th^ month* | 399.5 (250.5-591.0) | 380 (250-491) | 0.19 |
| *Median (IQR) CD4 count, 6^th^ month* | 424 (262-644) | 408 (246-493) | 0.16 |
| *Median (IQR) CD4 count, 12^th^ month* | 467 (281.0-627.5) | 421 (314-591) | 0.32 |
| *Median (IQR) CD4 count, 24^th^ month* | 550.5 (357.0-728.5) | 517 (397-615) | 0.32 |
| *Median (IQR) CD8 count, 1^st^ month* | 733 (503.5-1,122.5) | 700 (370-1,032) | 0.28 |
| *Median (IQR) CD8 count, 2^nd^ month* | 785.5 (517-1,223.5) | 823.5 (521-1,115) | 1.0 |
| *Median (IQR) CD8 count, 3^rd^ month* | 773.5 (592.5-1,234.0) | 856.5 (630-1,085) | 0.57 |
| *Median (IQR) CD8 count, 4^th^ month* | 825.5 (864.5-1,124.0) | 870 (677-1,121) | 0.20 |
| *Median (IQR) CD8 count, 5^th^ month* | 809.5 (564-1,107) | 858 (643-1,101) | 0.22 |
| *Median (IQR) CD8 count, 6^th^ month* | 794.5 (552.0-1,044,5) | 835 (685-1,141) | 0.13 |
| *Median (IQR) CD8 count, 12^th^ month* | 768 (525.0-1,064.5) | 831 (638-1,121) | 0.08 |
| *Median (IQR) CD8 count, 24^th^ month* | 806.5 (548.5-1,087.0) | 909.5 (572-1,133) | 0.48 |
| *Median (IQR) CD4/CD8 ratio, 1^st^ month* | 0.39 (0.2-0.62) | 0.29 (0.17-0.51) | 0.15 |
| *Median (IQR) CD4/CD8 ratio, 2^nd^ month* | 0.42 (0.25-0.67) | 0.32 (0.23-0.59) | 0.07 |
| *Median (IQR) CD4/CD8 ratio, 3^rd^ month* | 0.43 (0.26-0.72) | 0.36 (0.24-0.6) | 0.08 |
| *Median (IQR) CD4/CD8 ratio, 4^th^ month* | 0.49 (0.3-0.7) | 0.37 (0.26-0.63) | **0.02** |
| *Median (IQR) CD4/CD8 ratio, 5^th^ month* | 0.51 (0.32-0.78) | 0.4 (0.32-0.66) | **0.005** |
| *Median (IQR) CD4/CD8 ratio, 6^th^ month* | 0.57 (0.32-0.85) | 0.42 (0.31-0.71) | **0.006** |
| *Median (IQR) CD4/CD8 ratio, 12^th^ month* | 0.62 (0.38-0.89) | 0.49 (0.39-0.78) | **0.008** |
| *Median (IQR) CD4/CD8 ratio, 24^th^ month* | 0.85 (0.47-1.1) | 0.68 (0.55-0.91) | 0.09 |
| *CD4/CD8 ratio <0.45, 1^st^ month, n (%)* | 69 (57.5) | 48 (68.6) | 0.16 |
| *CD4/CD8 ratio <0.45, 2^nd^ month, n (%)* | 65 (54.2) | 48 (68.6) | **0.05** |
| *CD4/CD8 ratio <0.45, 3^rd^ month, n (%)* | 61 (50.8) | 47 (67.1) | **0.03** |
| *CD4/CD8 ratio <0.45, 4^th^ month, n (%)* | 47 (39.2) | 46 (65.7) | **<0.0001** |
| *CD4/CD8 ratio <0.45, 5^th^ month, n (%)* | 46 (38.3) | 45 (64.3) | **0.001** |
| *CD4/CD8 ratio <0.45, 6^th^ month, n (%)* | 43 (35.8) | 40 (57.1) | **0.004** |
| *CD4/CD8 ratio <0.45, 12^th^ month, n (%)* | 39 (32.5) | 33 (47.1) | **0.05** |
| *CD4/CD8 ratio <0.45, 24^th^ month, n (%)* | 34 (28.3) | 28 (40.0) | 0.10 |
| *CD4/CD8 ratio recovery ≥0.45*  *at 24^th^ month, n (%)* | 47 (68.1) | 30 (62.5) | 0.53 |
| *Immunological success at 24^th^ month, n (%)* | 99 (82.5) | 59 (84.3) | 0.75 |

**Table S2. Comparison between CD4+ and CD8+ T lymphocytes and CD4/CD8 ratio improvement in the 24 months following the start of cART in a population of HIV-positive HBcAb-positive/HBsAb-negative and HIV-positive HBcAb-positive/HBsAb-positive subjects**

|  | **HIV+/HBcAb+/HBsAb- (n= 28)** | **HIV+/HBcAb+/HBsAb+ (n= 42)** | **p-value** |
| --- | --- | --- | --- |
| *Median (IQR) CD4 count, 1^st^ month* | 195.5 (42.5-269.5) | 279.5 (155-477) | **0.02** |
| *Median (IQR) CD4 count, 2^nd^ month* | 210 (129-349.5) | 345.5 (207-429) | **0.04** |
| *Median (IQR) CD4 count, 3^rd^ month* | 265.5 (192.5-429.5) | 361 (214-475) | 0.14 |
| *Median (IQR) CD4 count, 4^th^ month* | 304 (162-472) | 412 (249-538) | 0.24 |
| *Median (IQR) CD4 count, 5^th^ month* | 316 (197-520) | 405.5 (302-469) | 0.26 |
| *Median (IQR) CD4 count, 6^th^ month* | 354.5 (234-469) | 430 (289-530) | 0.18 |
| *Median (IQR) CD4 count, 12^th^ month* | 374 (245-472) | 465 (342-598) | 0.13 |
| *Median (IQR) CD4 count, 24^th^ month* | 461.5 (391.5-467.5) | 540 (445-630) | 0.32 |
| *Median (IQR) CD8 count, 1^st^ month* | 572 (319-985) | 748.5 (497-1,037) | 0.30 |
| *Median (IQR) CD8 count, 2^nd^ month* | 776.5 (426-1,072) | 843.5 (635-1,145) | 0.57 |
| *Median (IQR) CD8 count, 3^rd^ month* | 856.5 (653-1,335.5) | 834 (602-1,042) | 0.81 |
| *Median (IQR) CD8 count, 4^th^ month* | 847 (633-1,067) | 878 (704-1,292) | 0.40 |
| *Median (IQR) CD8 count, 5^th^ month* | 1,022 (636-1,314) | 814.5 (643-1,061) | 0.18 |
| *Median (IQR) CD8 count, 6^th^ month* | 938.5 (694-1,159) | 825 (654-1,042) | 0.18 |
| *Median (IQR) CD8 count, 12^th^ month* | 888 (688-1,131) | 796 (621-1,106) | 0.21 |
| *Median (IQR) CD8 count, 24^th^ month* | 1,073 (697-1,158) | 754 (543-1,023) | 0.17 |
| *Median (IQR) CD4/CD8 ratio, 1^st^ month* | 0.27 (0.12-0.49) | 0.33 (0.2-0.6) | 0.08 |
| *Median (IQR) CD4/CD8 ratio, 2^nd^ month* | 0.32 (0.21-0.54) | 0.31 (0.23-0.64) | 0.15 |
| *Median (IQR) CD4/CD8 ratio, 3^rd^ month* | 0.35 (0.18-0.56) | 0.36 (0.24-0.62) | 0.16 |
| *Median (IQR) CD4/CD8 ratio, 4^th^ month* | 0.37 (0.27-0.64) | 0.4 (0.26-0.63) | **0.03** |
| *Median (IQR) CD4/CD8 ratio, 5^th^ month* | 0.39 (0.28-0.63) | 0.43 (0.32-0.68) | **0.009** |
| *Median (IQR) D4/CD8 ratio, 6^th^ month* | 0.37 (0.28-0.55) | 0.49 (0.33-0.72) | **0.008** |
| *Median (IQR) CD4/CD8 ratio, 12^th^ month* | 0.48 (0.37-0.57) | 0.59 (0.39-0.96) | **0.01** |
| *Median (IQR) CD4/CD8 ratio, 24^th^ month* | 0.6 (0.46-0.74) | 0.69 (0.58-1.01) | 0.09 |
| *CD4/CD8 ratio <0.45, 1^st^ month, n (%)* | 20 (71.4) | 28 (66.7) | 0.29 |
| *CD4/CD8 ratio <0.45, 2^nd^ month, n (%)* | 19 (67.9) | 29 (69.1) | 0.15 |
| *CD4/CD8 ratio <0.45, 3^rd^ month, n (%)* | 21 (75.0) | 26 (61.9) | **0.05** |
| *CD4/CD8 ratio <0.45, 4^th^ month, n (%)* | 20 (71.4) | 26 (61.9) | **0.001** |
| *CD4/CD8 ratio <0.45, 5^th^ month, n (%)* | 20 (71.4) | 25 (59.5) | **0.002** |
| *CD4/CD8 ratio <0.45, 6^th^ month, n (%)* | 19 (67.9) | 21 (50.0) | **0.006** |
| *CD4/CD8 ratio <0.45, 12^th^ month, n (%)* | 17 (60.7) | 16 (38.1) | **0.02** |
| *CD4/CD8 ratio <0.45, 24^th^ month, n (%)* | 12 (42.9) | 16 (38.1) | 0.23 |
| *CD4/CD8 ratio recovery ≥0.45 at 24^th^ month, n (%)** | 11 (39,2) | 19 (45,2) | 0.53 |
| *Immunological success at 24^th^ month, n (%)* | 24 (85.7) | 35 (83.3) | 0.96 |
